# A Textbook of Pathology and Pathological Anatomy

**Published:** 1903-07

**Authors:** 


A TEXTBOOK OF PATHOLOGY AND PATHOLOGICAL ANATOMY. By DR. HANS SCHMAUS, Extraordinary Professor and First Assistant in the Pathological Institute, Munich. Translated from the sixth German edition by A. E. THAYER, M. D., Instructor in Pathology in Cornell University Medical College, New York. Edited with additions by James Ewing, M. D., Professor of Pathology in Cornell University Medical College, New York. Octavo, pp. 614. Illustrated with 351 engravings, including 35 colored insert plates. Lea Brothers & Co., Philadelphia and New York. 1902. (Cloth, $4.00 net.)

The reputation of Professor Schmaus of Munich, as a pathologist and as a teacher of pathological anatomy, is so great that his work on pathology easily earned recognition, and soon won the foremost place in Germany. The reason for its great popularity, six editions in the original, having already been demanded, is easily discernable on examination of the book. It is so well adapted to the needs of the student and practitioner that an English translation was demanded and Professor Ewing, whose work on the blood is so well known, was chosen to edit the American edition.

The author divides his work into two parts; Part I. being devoted to general pathology, and Part II. to special pathology. The usual processes as the degenerations, the infiltrations, disorders of circulation, inflammations, deformities and the parasites are minutely described in part one.

The special pathology of the various organs of the body are carefully treated, and especial attention given to the diseases of the brain and spinal cord. These chapters are, perhaps, the best in the book and are beautifully and elaborately illustrated. The color plates are many and convey to the eye what the text so well portrays. These plates are important also, because they show the characteristic staining of the different methods. Particularly good is the colored plate illustrating syringomyelia and hydromyelia; the one stained with ammonia carmine, the other with Weigert's stain. The translation has been rendered in a graceful, easy style.

